# First record of *Neobenedenia girellae* (Monogenea: Capsalidae) as the cause of mortality of yellow snappers, *Lutjanus argentiventris*, farmed in artisanal cages in the Urías Estuary, Mazatlán, Mexico

**DOI:** 10.2478/helm-2026-0008

**Published:** 2026-06-15

**Authors:** J. A. G. LÓPEZ-CESEÑA, L. ANDRADE-GÓMEZ, B. MENDOZA-GARFIAS, G. PÉREZ-PONCE DE LEÓN, M. I. GRANO-MALDONADO

**Affiliations:** 1 Facultad de Ciencias del Mar, Universidad Autónoma de Sinaloa, Mazatlán, Sinaloa, México; 2 Escuela Nacional de Estudios Superiores Unidad Mérida, Universidad Nacional Autónoma de México, Mérida, Yucatán, México; 3 Instituto de Biología, Universidad Nacional Autónoma de México, Ciudad de México

**Keywords:** capsalid, Monogenea, aquaculture, Sinaloa, Mexico

## Abstract

A parasitic outbreak of capsalid monogeneans occurred in May 2023 in artisanal cages where yellow snappers (*Lutjanus argentiventris*) are farmed. Cages are set at the entrance to the Urías Estuary in Mazatlán, northwestern Mexico. The outbreak resulted in the loss of 8,890 fish from an initial stock of 10,000. Twenty fish specimens were examined to determine the cause of death. Massive tissue damage was observed in the hosts’ skin, and a severe infection with capsalid monogeneans was detected. The capsalid was initially identified morphologically as a member of the genus *Neobenedenia*; however, due to long-standing taxonomic confusion between *N. girellae* and *N. melleni*, we further corroborated the identification using ribosomal (28S rRNA) and mitochondrial (cytb) DNA sequences, analyzing sequence divergence values and phylogenetic interrelationships. Photomicrographs of processed specimens, as well as scanning electron photomicrographs, were obtained. The monogenean was identified as *Neobenedenia girellae* and is reported for the first time as a parasite of farmed *L. argentiventris*. The monogenean reached very high infection levels, with a prevalence of 100 ^¢^%, and a mean intensity of 68.2 parasites per infected host. To more accurately describe the effect of this highly pathogenic monogenean on the host, we assessed the body condition factor of the hosts (1.22 ± 0.05). We compared it with the standard value reported in the literature.

## Introduction

Members of the family Capsalidae are recognized as among the most important fish parasites for fisheries and aquaculture worldwide (Brazenor *et al*., 2018a; Tedesco *et al*., 2021). These monogeneans cause skin damage through mechanical fixation of the hooks, leading to dermal erosion, inflammation, and, most importantly, allowing the development of secondary pathogens (Whittington, 2012; Trujillo-González *et al.,* 2015). These infections may result in catastrophic losses of fish populations and aquaculture production. Under some conditions, capsalids have developed tolerance to chemical treatments (Ogawa, 2002, 2015), making the control of parasites in captivity challenging. In Mazatlán Bay, in Northwestern Mexico, at least six species of capsalids (*Capsala laevis,* Verrill 1875; *Capsala pricei,* Hidalgo-Escalante 1958; *Capsaloides hoffmannae,* Lamothe-Argumedo 1996; *Capsaloides sinuatus,* Goto 1894; *Encotyllabe pagrosomi,* MacCallum 1917; *Neobenedenia melleni,* MacCallum 1927) have been recorded parasitizing commercially important fish such as spearfish and marlin (Istiophoridae), snappers (Lutjanidae), and pufferfish (Tetraodontidae). One of these capsalids, *Neobenedenia melleni,* has been reported in the locality as a parasite of the bullseye puffer, *Sphoeroides annulatus* (Jenyns, 1842) (see Grano-Maldonado & Pérez-Ponce de León, 2023 and references therein). Additional records of this highly pathogenic monogenean have been made, although no effort was made to identify them to the species level. For example, Morales-Serna *et al*. (2025) reported an outbreak in the ocean tank at the Mazatlán aquarium caused by *Neobenedenia* sp., which primarily affected three fish species: the Pacific crevalle jack (*Caranx caninus*), the Pacific spadefish (*Chaetodipterus zonatus*), and the Colorado snapper (*Lutjanus colorado*)*.*

Distinguishing among *Neobenedenia* species is challenging (Whittington *et al*., 2004). In particular, there is no straightforward way to distinguish morphologically *N. melleni* from *N. girellae*. These species were historically synonymized (Whittington & Horton, 1996), a classification that has since been rejected but remains a source of taxonomic confusion. However, based on the findings of Whittington *et al*. (2004) and Perkins *et al*. (2009) and following molecular evidence from multiple countries, Brazenor *et al*. (2018a) first proposed *N. girellae*—rather than *N. melleni*—as the primary parasitic monogenean in worldwide aquaculture, noting that the two are cryptic species. Building on this, Tedesco *et al*. (2021) emphasized that, because *N. girellae* morphology varies with host species and environmental conditions, an integrated approach combining morphology and molecular methods is essential to accurately differentiate these morphologically indistinguishable taxa.

A paper describing an allegedly new species of *Neobenedenia, N. cibnorensis* Valles-Vega, Pérez-Urbiola & Ascencio, 2025, was recently published, parasitizing the Alamo Jack, *Seriola rivoliana*, from La Paz, Baja California Sur (Valles-Vega *et al*., 2025). However, as demonstrated in the present study, the analyses and interpretations of phylogenetic trees and genetic divergence values in Valles-Vega *et al.* (2025) are flawed, and the description of a new species was mistaken; the sequences of these specimens actually correspond to *N. girellae.*

*Neobenedenia girellae* exhibit low host specificity, possess a large infectivity capacity, and has been recorded in approximately 30 host species allocated in families such as Carangidae, Cirrhitidae, Coryphaenidae, Labridae, Latidae, Microdesmidae, Pleuronectidae, Pseudochromidae, Rachycentridae, and Serranidae (Hirayama *et al*., 2009; Brazenor *et al*., 2018 a,b; Tedesco *et al*., 2021); they exhibit a wide distributional range across the world and have been a matter of concern because they have been responsible for causing disease, mortality, and economic losses in marine farmed fish worldwide (Whittington, 2012; Hirazawa *et al*., 2016; Brazenor *et al*., 2018a,b).

Lutjanids (snappers) are commercially important marine fish distributed worldwide; in some regions, they have been overexploited (Amorim *et al*., 2019). Because of their high market prices and limited harvest from wild stocks, considerable interest in farming a variety of snapper species has developed (Ibarra-Castro *et al.,* 2020). In particular, some areas of northwestern Mexico have been considered as potential sites for snapper farming (Muhlia-Melo *et al*., 2003). In recent years, a semi-intensive artisanal mariculture system has been implemented and developed in the port of Mazatlán, specifically in the Urías estuary, whose entrance serves as a fishing port, shipyard, and port facility. Each year, juvenile specimens of various snapper species are captured in the estuary, placed in cages, fed to promote growth, and harvested when they reach commercial size. In May 2023, an outbreak occurred in cages containing yellowtail snapper (*Lutjanus argentiventris*), resulting in the loss of 8,890 fish from an initial population of 10,000. A significant infection with monogenean capsalids of the genus *Neobenedenia* was found to have caused the fish’s death. The main objective of this study was to identify the species of *Neobenedenia* that caused the outbreak by using morphological and molecular tools; additionally, the prevalence and mean intensity of infection, as well as the condition factor of the hosts, were also reported as an assessment of the effects of the parasite on their hosts.

## Materials and Methods

### Source of hosts and parasites, and a morphological study

Twenty moribund individuals of *Lutjanus argentiventris* from the artisanal cages where the outbreak occurred in May 2023 were taken in a cooler with original water from the farm to the Facultad de Ciencias del Mar (FACIMAR) of the Universidad Autónoma de Sinaloa. The Urías Estuary is located in the southeast Gulf of California, in the Mexican Pacific, south of the city of Mazatlán. Individual fish were sacrificed according to the Mexican Law NOM-033-SAG/ZOO-2014, washed with fresh tap water, and passed through a 20 μm mesh filter to recover any dislodged parasites. The body surface, mouth, gill chamber, and gills were also examined. Monogeneans were removed using needles and placed in Petri dishes with 8.5 % saline. Specimens were fixed in hot (near-boiling) water and placed in vials containing 100 % ethanol for morphological and molecular analyses. For morphology, seven specimens were stained with Mayer’s paracarmine and Gomori’s trichrome, cleared in methyl salicylate, and mounted on permanent slides with Canada balsam. Voucher specimens were deposited at the Colección Nacional de Helmintos (CNHE), Mexico City, under accession number CNHE 12397. For scanning electron microscopy, two specimens were dehydrated in a graded ethanol series, critical point dried, sputter-coated with gold and examined with a Hitachi Stereoscan Model SU1510 scanning electron microscope at 10 kV at the Laboratorio de Microscopía y Fotografía de la Biodiversidad (LANABIO), Instituto de Biología, Universidad Nacional Autónoma de México (UNAM).

### Molecular analyses

Three specimens were processed for molecular analyses to aid in species identification. The digestion, amplification, and sequencing protocols followed those of Andrade-Gómez *et al*. (2023) and Brazenor *et al*. (2018a). Two molecular markers were sequenced. The D1-D3 domains of the large subunit of the ribosomal DNA (28S) was amplified using the primers 391 (5’-AGCG-GAGGAAAAGAAACTAA-3’) and 536 (5’-CAGCTATCCTGAGG-GAAAC-3’) (García-Varela & Nadler, 2005), and the mitochondrial Cytochrome b (cytb) gene was amplified using the primers M1676 (5’-TGAGTTATTATTGATGTAGAGG-3’) and M1677 (5’-AAAATAT-CAKTCAGGCTTWA-3’) (Brazenor *et al*., 2018a). Sequences were assembled and edited using Geneious v7 (Kearse *et al*., 2012). The two datasets were built, including newly obtained sequences and sequences available in GenBank for *N. girellae* and other congeneric species; *Benedenia* spp. was used as the outgroup for rooting each tree. When downloading cytb sequences for a proposed new species, *Neobenedenia cibnorensis* (OQ988164, OR022087-89, OR225614, OR192968, OR184924-25, referred as *Neobenedenia* sp. in GenBank), we noticed that these sequences were reversed. Therefore, we used the complementary reverse complements of these sequences and generated a new alignment to construct phylogenetic trees and estimate pairwise genetic divergence values. The best-fit molecular evolution model for each dataset was estimated with the jModelTest 0.1.1 program (Posada, 2008) with the Akaike information criterion (AIC). The best model for 28S was GTR + I, and for cytb was GTR + I + G. Phylogenetic analysis was conducted using Maximum Likelihood (ML) with RAxML version 7.0.4 (Silvestro & Michalak, 2011) through the interface: Cyberinfrastructure for Phylogenetic Research Science Gateway v3.3 (CIPRES) (Miller *et al*., 2010). A 1000 bootstrap replicates were run to support each node in the phylogenetic analyses. A phylogenetic tree was drawn in FigTree v.1.3.1 (Rambaut, 2012). Genetic divergence among taxa was estimated using uncorrected ‘p’ distances with MEGA version 6 (Tamura *et al*., 2013).

### Infection parameters

All examined fish were weighed and measured, and their sex was determined by gonadal morphology. With these data, the condition factor (CF) was determined using the following equation: CF = ((total weight (g)/total length^3^ (cm)) *100) (Le Cren, 1951). Ecological parameters were estimated following Bush *et al*. (1997).

## Results

**Fig. 1.**
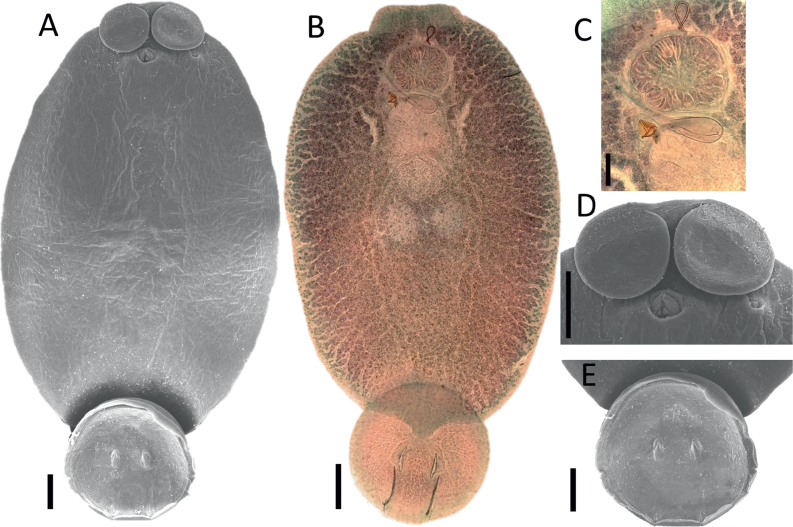
*Neobedenia girellae*, A) Photomicrograph of scanning electron microscopy (SEM) of an adult, ventral view; B) Photomicrograph of an adult specimen stained with Mayer’s paracarmine, dorsal view; C) Detail of the anterior end of a stained specimen showing a filamented egg; D) SEM of anterior end showing the attachment organs; E) SEM of posterior end showing the structure of the haptor. Scale bar = 200 μm

**Fig. 2.**
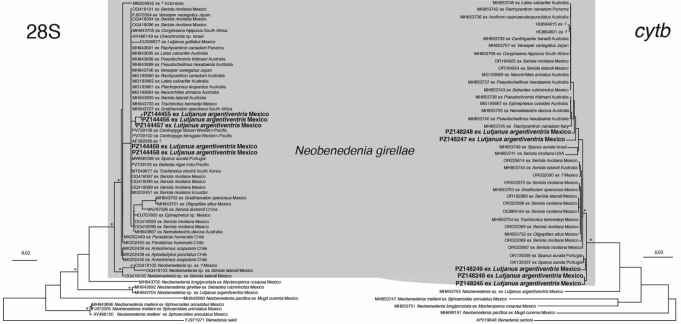
Maximum-likelihood phylogenetic trees (28S and cytb) showing the phylogenetic position of *Neobenedenia girellae* isolates from *Lutjanus argentiventris* in the Estero Urías, Mazatlán. Asterisks above nodes represent bootstrap support values higher than 80.

The examined fish showed significant damage to its body surface. Skin lacerations were visible, some with heavy bleeding and signs of exophthalmia; these lesions were produced by a large number of capsalid monogeneans infecting these fish. The monogeneans were morphologically identified as belonging to the genus *Neobenedenia* by having two almost circular and unlobed anterior attachment organs and a well-developed haptor possessing two pairs of accessory sclerites and marginal hooklets (Fig. 1). Characteristically, species in the genus possess an extension of the anterior body margin between the attachment organs along the midline of the body (Fig. 1). DNA sequences of the 28S rRNA gene and the mitochondrial gene Cytochrome b (cytb) allowed the identification of *Neobenedenia* as *N. girellae*. In both phylogenetic trees, the newly sequenced individuals nested in a clade with isolates of *N. girellae* from different host species and geographical locations, and these relationships were supported by a high bootstrap value (Fig. 2). The genetic divergence estimated among isolates of *N. girellae* varied from 0 to 3.5 % for the 28S rRNA gene, and from 0 to 3.7 % for cytb. Instead, the interspecific genetic divergence between *N. girellae* and all other congeners for which sequence data are available ranged from 5.7 to 12.3 % for 28S, whereas for cytb, it ranged from 9.7 to 16.6 %. In particular, sequences of *N. girellae* differed from those of *N. melleni* from 7.4 to 9 % for 28S and 13 – 14.9 % for cytb. Furthermore, the topology of the 28S rRNA gene shows that these two species are not sister taxa (Fig. 2).

### Infection parameters

The condition factor was 1.22 ± 0.05, with a range of 1.14 – 1.32. Twenty fish were examined, and 1,364 specimens of *N. girellae* were found. The prevalence of infection was 100 %, with a mean intensity of 68.2 parasites per infected host.

## Discussion

The practice of identifying parasite species infecting fish populations, either in the wild or under aquacultural conditions, is fundamental. Furthermore, establishing the diversity and ecological attributes, such as prevalence and mean intensity of infection of the parasite fauna fish, is very important to understand host-parasite associations, life cycles and epidemiology dynamics, especially when samples are compared at different spatial and time scales, i.e., when comparing with other fish species in the same ecosystem, or when comparing the same fish species through a time series.

Additionally, in fish populations subjected to aquaculture practices, precisely identifying the parasite species causing disease is essential for implementing prophylactic or control measures; some methods may be specific to a particular parasite species. For instance, some studies have approached the effective control of *N. girellae* infestations in juveniles of *Seriola dumerili* under controlled conditions using praziquantel (Quirós-Pozo *et al*., 2025); other studies have evaluated, for example, the phytotherapeutic effects of essential oils, and the chemotherapeutics such as formalin, potassium permanganate, hydrogen peroxide, and salinity on *Neobenedenia melleni* infecting cultivated *Mugil liza*. However, no studies have been conducted to test the efficacy of treatments for controlling fish infections in a comparative framework across different *Neobenedenia* species*.* In some cases, as in *N. girellae,* it has been determined that acquired protection against challenge infections can occur in naturally infected fish (Bondad-Reantaso *et al*., 1995), which, according to Buchman and Bresiani (2006), may suggest an immunoprophylactic strategy. Whether the same response occurs in *N. melleni* has not been determined. The latter illustrates one of the reasons for identifying the species of the parasite causing a disease; as discussed earlier, distinguishing two morphologically very similar species, such as *N. girellae* and *N. melleni*, is challenging, especially because *N. girellae* varies with host fish species and environmental conditions (Brazenor *et al*., 2018a). As a result, DNA sequences are beneficial for distinguishing *N. girellae* from *N. melleni* (Tedesco *et al*., 2021). We believe that all studies on *Neobenedenia*, particularly when it can be suspected that the species corresponds either to *N. melleni* or *N. girellae,* should use DNA sequences to achieve a more reliable identification, mainly because these monogeneans are widely distributed and are responsible for causing disease, mortality, and economic losses in marine farmed fish worldwide (Whittington, 2012; Hirazawa *et al*., 2016; Brazenor *et al*., 2018a,b).

A further note on the importance of determining parasite species to the lowest taxonomic level was illustrated by Paredes-Trujillo *et al*. (2022). These authors detected infections with a capsalid monogenean in 40 species of marine ornamental fish imported for commercial purposes to a public aquarium in the Mexican state of Yucatan. Using partial sequences of the 28S rRNA gene, these authors corroborated that all the fish were infected with *N. girellae*; this is relevant information for aquarists and aquaculturists because this monogenean is highly pathogenic to fish kept under confined conditions.

The proper use and analysis of sequence data are also essential for accurate species identification. Valles-Vega *et al*. (2025) proposed a new species of *Neobenedenia*, *N. cibnorensis,* as a parasite of a carangid fish in Baja California Sur, Mexico. The authors based the distinction of the allegedly new species from *N. girellae* and *N. melleni* mainly on the divergence level of the mitochondrial gene cytb, set at 58 % in their analysis relative to all other *Neobenedenia* spp. However, we noticed that their cytb sequences were reversed, and this clearly affected their results and interpretations. In the present study, sequences of that species were nested within a clade with other *N. girellae* sequences, and the genetic divergence among all isolates was 73.7 %. Therefore, we consider *N. cibnorensis* as a junior synonym of *N. girellae.* Monogeneans are considered lethal parasites in aquaculture. Members of the genus *Neobenedenia* are notable for producing viable eggs in isolation for three consecutive generations, totaling up to 3,300 eggs over 17 days (Dinh-Hoai & Hutson, 2014). This feature of *Neobenedenia* is probably the cause of the success in generating an outbreak like the one we report herein in yellow snappers intended for growth in artisanal cages at the Urías Estuary. In this case, for the semi-intensive, artisanal system, juvenile yellow snappers are captured from the Urías Estuary, placed in cages near the coast, fed to promote growth, and harvested once they reach commercial size. Clearly, fish were introduced with some *Neobenedenia* specimens, which reproduced and took advantage of the fish’s high density in the cages. This case is not uncommon, as the hazards of monogeneans to fsh kept in confnement have previously been discussed (Thoney & Hargis, 1991). It has been shown that, once in fsh, *N. girellae* in particular causes severe effects (Hirayama *et al*., 2009; Hirazawa *et al*., 2016).

Fish condition factor (CF) values can vary across species, life stages, and environmental conditions (Abdul *et al.,* 2017). Our results indicated that the fish’s condition factor in yellow snappers was 1.22 ± 0.05, with a range of 1.14 – 1.32, which is below the typical range for the species (1.35 – 1.45) according to a previous study conducted by Lucano-Ramírez *et al.* (2014), which is the only available bibliographical source to compare. Usually, a low CF in fish signals stress, malnutrition, or illness (Cifuentes *et al*., 2012), and parasite burden directly influences CF (Özer *et al*., 2016). In this study, the lower CF (1.14) may be linked to the presence of the monogenean *Neobenedenia girellae*, which caused mortality in yellow snappers (*Lutjanus argentiventris*) farmed in artisanal cages in the Urías Estuary, Mazatlán, on the northwest Mexican Pacific.
